# Allele frequency of antiretroviral host factor TRIMCyp in wild-caught cynomolgus macaques (*Macaca fascicularis*)

**DOI:** 10.3389/fmicb.2012.00314

**Published:** 2012-08-30

**Authors:** Akatsuki Saito, Yoshi Kawamoto, Atsunori Higashino, Tomoyuki Yoshida, Tomoko Ikoma, Yuriko Suzaki, Yasushi Ami, Tatsuo Shioda, Emi E. Nakayama, Hirofumi Akari

**Affiliations:** ^1^Primate Research Institute, Kyoto UniversityInuyama, Japan; ^2^Division of Experimental Animal Research, National Institute of Infectious DiseasesShinjuku-ku, Japan; ^3^Department of Viral Infections, Research Institute for Microbial Diseases, Osaka UniversitySuita, Japan

**Keywords:** cynomolgus monkey, TRIM5α, TRIMCyp, genetic diversity, host factor

## Abstract

A recent study showed that the frequency of an antiretroviral factor *TRIM5* gene-derived isoform, TRIMCyp, in cynomolgus macaques (*Macaca fascicularis*) varies widely according to the particular habitat examined. However, whether the findings actually reflect the prevalence of TRIMCyp in wild cynomolgus macaques is still uncertain because the previous data were obtained with captive monkeys in breeding and rearing facilities. Here, we characterized the *TRIM5* gene in cynomolgus macaques captured in the wild, and found that the frequency of the TRIMCyp allele was comparable to those in captive monkeys. This suggests that the previous results with captive monkeys do indeed reflect the natural allele frequency and that breeding and rearing facilities may not affect the frequency of *TRIM5* alleles. Interestingly, the prevalence of a minor haplotype of TRIMCyp in wild macaques from the Philippines was significantly lower than in captive ones, suggesting that it is advantageous for wild monkeys to possess the major haplotype of TRIMCyp. Overall, our results add to our understanding of the geographic and genetic prevalence of cynomolgus macaque TRIMCyp.

## Introduction

In 2004, TRIM5-Cyclophilin A (CypA) chimeric protein, referred to as TRIMCyp, was first identified in owl monkeys (*Aotus trivirgatus*), which belongs to New World monkeys (NWMs) (Sayah et al., 2004). The discovery of TRIMCyp in owl monkeys explains the novel post-entry restriction of human immunodeficiency virus type 1 (HIV-1), which is uniquely seen in owl monkey-derived cells but not in other NWM-derived cells. Owl monkey TRIMCyp is derived from LINE-1-mediated retrotransposition of CypA cDNA into the region between *TRIM5* exons 7 and 8. On the other hand, the strong post-entry restriction of HIV-1 in Old World monkey (OWM)-derived cells was thought to be dependent on a TRIM5α-mediated mechanism (Stremlau et al., 2004; Nakayama and Shioda, 2010). Interestingly, among OWMs, pig-tailed macaques (*Macaca nemestrina*; hereafter denoted as PMs) uniquely show higher susceptibility to HIV-1 infection when compared with other OWMs (Agy et al., 1992). However, the mechanism underlying this higher susceptibility was unclear. Thereafter, it was found that PMs exclusively have the TRIMCyp genotype, which is a strong genetic determinant of their susceptibility to HIV-1 infection (Liao et al., 2007; Brennan et al., 2008; Virgen et al., 2008). Subsequently, TRIMCyp was also discovered in rhesus macaques (*Macaca mulatta*; hereafter denoted as RMs) and cynomolgus macaques (*Macaca fascicularis*; hereafter denoted as CMs) (Brennan et al., 2008; Newman et al., 2008; Wilson et al., 2008).

TRIMCyp is an alternatively spliced isoform of the *TRIM5* gene in which the PRYSPRY domain of TRIM5α is replaced with a retrotransposed *CypA* gene. Unlike owl monkey TRIMCyp, the *CypA* gene in OWM TRIMCyp, is inserted in the 3′-untranslated region (UTR) of the *TRIM5* gene. The retrotransposition of the CypA sequence is concomitant with a single nucleotide polymorphism (SNP) at the exon 7 splice acceptor site; this leads to skipping of exons 7 and 8 encoding the PRYSPRY domain and splicing to the inserted *CypA* gene (Johnson and Sawyer, 2009). Thus, the presence or absence of the CypA sequence in the 3′ UTR leads to expression of TRIMCyp or TRIM5α (Nakayama and Shioda, 2012).

Current data suggest that PMs exclusively express TRIMCyp and not TRIM5α. In the case of RMs, the frequency of TRIMCyp in Indian RM was approximately 25%, while it was not found in the Chinese RM population (Wilson et al., 2008). In addition, we observed that the frequency of TRIMCyp in Burmese RM was approximately 10% (unpublished data), suggesting a geographical deviation in the frequency of RM TRIMCyp. In the case of CM, we and other groups reported that TRIMCyp is present at higher frequency when compared with RM (De Groot et al., 2011; Dietrich et al., 2011; Saito et al., 2012). Interestingly, we and other groups found a geographical deviation in the frequency of TRIMCyp in CM (Dietrich et al., 2011; Berry et al., 2012; Saito et al., 2012). In particular, we showed that the frequency of TRIMCyp in the Philippine population was higher than that in Indonesian and Malaysian populations. Dietrich et al. also reported that the frequency of TRIMCyp in the Philippine population was higher than that in Indonesia, Indochina, and Mauritian populations (Dietrich et al., 2011). Moreover, they claimed that the frequency of TRIMCyp in Indonesian CMs was higher than that of Indochina and Mauritian populations. However, all these analyses were performed with captive monkeys in breeding and rearing facilities. Therefore, these results may not reflect the natural gene frequencies. For instance, a small number of animals of a certain genotype introduced into facilities may affect the frequency of TRIMCyp via the founder effect. Furthermore, breeding policies may lead to a deviation of specific genotype. Hence, in order to understand the prevalence of TRIMCyp in CM precisely, it is necessary to analyze the frequency of TRIMCyp in wild CM. Therefore, in the present study, we sought to determine the geographic and genetic diversity of the *TRIM5* gene in wild-caught CM.

## Materials and methods

### Sample collection

Blood samples from the wild-caught CMs, which had been cryopreserved for veterinary and microbiological examination as quarantine, were used in this study. These animals had been imported in the 1970's from the Philippines, Malaysia, and Indonesia to Japan as the founders of a breeding colony. These animals were directly sent to Japan without breeding in these countries.

### Determination of *TRIM5* genotype

The genotyping of *TRIM5* gene was performed as described previously with slight modifications (Saito et al., 2012). Briefly, the genomic DNA was extracted from frozen blood samples of 88 CMs with a QIAamp DNA Blood Mini kit (*Qiagen*, *Tokyo, Japan*). The genomic DNA was amplified by PCR using Ex Taq HS (TaKaRa, *Otsu*, *Japan*) with TC forward (5′-TGA CTC TGT GCT CAC CAA GCT CTT G-3′) and TC reverse (5′-ACC CTA CTA TGC AAT AAA ACA TTA G-3′) primers as described by Wilson et al. (2008). After amplification, PCR products were visualized on a 1% agarose gel stained with ethidium bromide.

### Sequencing of the CypA domain of TRIMCyp

Amplified products of the CypA domain from 44 TRIMCyp homozygotes and 21 TRIMCyp/TRIM5α heterozygotes were purified using Wizard SV Gel and PCR Clean-Up System (*Promega*, *Tokyo, Japan*) and then subjected to direct sequencing using primer pairs of MfasCypA_F (5′-CAA CCC TAC CGT GTT CTT CG-3′) and MfasCypA_R (5′-TCG AGT TGT CCA CAG TCA GC-3′). Sequencing products were analyzed on a 3130xl Genetic Analyzer (*Applied Biosystems*, *Tokyo*, *Japan*).

## Results

### Higher frequency of TRIMCyp in a wild philippine population as compared to indonesian and malaysian populations

**Figure 1:**
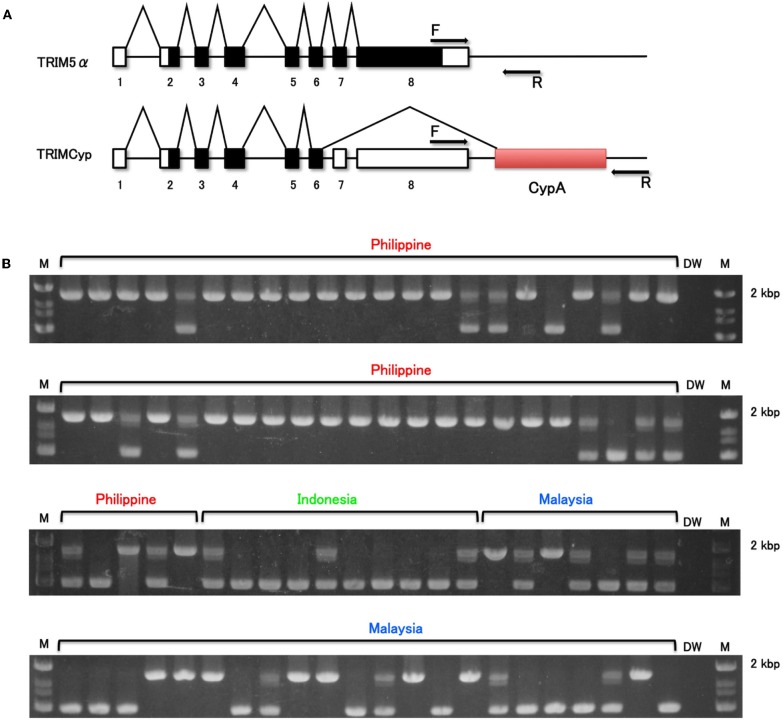
**Determination of *CypA* insertion. (A)** Diagram indicating splicing of TRIM5α or TRIMCyp. Noncoding and coding exons and CypA sequences are shown in white, black, and shaded white, respectively. F and R denote forward and reverse primers used in this study, respectively. **(B)** The genomic DNA was extracted from whole blood. To test for CypA insertion, the 3′ region of the *TRIM5* gene was amplified by PCR with primers spanning the 3′ UTR and the putative *CypA* insertion. M and DW denote DNA molecular weight standard marker and water control, respectively.

We first analyzed the frequency of TRIMCyp in these wild-caught animals. The PCR-based assay performed here was designed to differentiate between the presence and absence of the CypA insertion (Figure 1A). The electrophoretic pattern of PCR products is shown in Figure 1B. The upper bands indicate TRIMCyp, while the lower bands indicate TRIM5α. A heterozygote is expected to possess both bands. As summarized in Table 1, we found that the 35 of the 49 Philippine CMs were homozygous for TRIMCyp, 11 were heterozygous, and 3 were homozygous for TRIM5α. In the case of Malaysian CM, 11 of the 29 animals were homozygous for TRIMCyp, 8 were heterozygous, and 10 were homozygous for TRIM5α. Finally, in the case of Indonesian CMs, none of the 10 animals were homozygous for TRIMCyp, 3 were heterozygous, and 7 were homozygous for TRIM5α. The calculated frequency of TRIMCyp in these populations was 82.7%, 48.3%, and 15.0%, respectively. Statistical analysis revealed that the frequency of TRIMCyp in the Philippine population was significantly higher than that in the Indonesian and Malaysian populations.

**Table 1:** **Frequency of TRIMCyp alleles in wild Philippine, Malaysian, and Indonesian populations**.

**Country**	**Origin of sample**	**#animals**	**Genotype (# animals)**			**Allele frequency**		**Citation**
			**TRIM5α homozygote**	**heterozygote**	**TRIMCyp homozygote**	**% TRIM5**α	**% TRIMCyp**	
Philippines	Wild-caught	49	3	11	35	17.3	82.7	This study
Philippines	Captive	46	1	10	35	13.0	87.0	Saito et al., 2012
Philippines	Captive	4	0	0	4	0	100	Dietrich et al., 2011
Malaysia	Wild-caught	29	11	8	10	51.7	48.3	This study
Malaysia	Captive	47	11	26	10	51.1	48.9	Saito et al., 2012
Indonesia	Wild-caught	10	7	3	0	85.0	15.0	This study
Indonesia	Captive	33	13	17	3	65.2	34.8	Saito et al., 2012
Indonesia	Captive	18	3	10	5	44.4	55.6	Dietrich et al., 2011

### Difference in the haplotype frequency of TRIMCyp between wild and captive philippine populations

Others and we have recently demonstrated the presence of several haplotypes in TRIMCyp of captive CM (Dietrich et al., 2011; Saito et al., 2012). Specifically, the major haplotype in which amino acid residues at positions 369 (Cyp66) and 446 (Cyp143) are aspartic acid (D) and lysine (K) is abundant in captive-CM TRIMCyp alleles [denoted as TRIMCyp-major (DK)]. In addition, the minor haplotype encoding asparagine (N) and glutamic acid (E) at positions 369 (Cyp66) and 446 (Cyp143) is also present [denoted as TRIMCyp-minor (NE)].

In this study, we further investigated the haplotypes of TRIMCyp in the wild-caught CM and compared the frequency of each haplotype in these animals with those reared in captivity. We found that although both haplotypes were present in wild-caught CM, the frequency of TRIMCyp-minor (NE) in wild Filipino CM was much less than that in captive Filipino CM (1.2% versus 14.3% of TRIMCyp; *p* < 0.01) (Table 2). By contrast, the frequency of TRIMCyp-minor (NE) in wild Malaysian CM was comparable to that in captive Malaysian CM (10.7% versus 11.1% of TRIMCyp; *p* > 0.05). In the case of wild Indonesian CM, all animals analyzed here possess TRIMCyp-major (DK), although the size of samples was too small to determine whether this was significant. Thus, the precise frequency of each haplotype in wild Indonesian CM is unclear.

**Table 2:** **Frequencies of DK and NE haplotypes in TRIMCyps of wild CM**.

**Country**	**Origin of sample**	**#animals**	**Genotype (# chromosomes)**				**Frequency**		**Citation**
			**TRIM5α/TRIMCyp heterozygote^a^**		**TRIMCyp homozygote^b^**		**% DK**	**% NE**	
			**DK**	**NE**	**DK**	**NE**			
Philippines	Wild-caught	46	10	1	70	0	98.8	1.2	This study
Philippines	Captive	28	6	1	36	6	85.7	14.3	Saito et al., 2012
Malaysia	Wild-caught	18	7	1	18	2	89.3	10.7	This study
Malaysia	Captive	21	14	1	10	2	88.9	11.1	Saito et al., 2012
Indonesia	Wild-caught	3	3	0	0	0	100	0	This study
Indonesia	Captive	15	12	0	4	2	88.9	11.1	Saito et al., 2012

a Haplotypes were determined by direct sequencing of the PCR products.

b Haplotypes were inferred by the Maximum-Likelihood estimation using the results of direct sequencing of the PCR products.

## Discussion

In the present study, we analyzed the incidence of TRIMCyp in wild-caught animals and found that its frequency was comparable to that in captive animals (Table 1). Although blood samples from other regions were unavailable, it is reasonable to assume that the equivalence in the frequency of TRIMCyp between captive and wild-caught CM in other regions may have a similar tendency. Interestingly, we also found that the frequency of the TRIMCyp-minor (NE) haplotype in wild CM was lower than that in captive CM in the case of the Filipino population, but not in the case of the Malaysian population (Table 2). Although the reason for this discrepancy remains to be elucidated, we speculate that it might be hazardous for wild Filipino CM to possess TRIMCyp-minor (NE), as it may render them susceptible to TRIMCyp-minor (NE)-resistant pathogens present in the Philippines, but not in Malaysia. Based on this hypothesis, wild Filipino CM might be forced to expand TRIMCyp-major (DK) in order to counteract invasions from such pathogens. Conversely, weaker attacks, if any, from these pathogens in the breeding and rearing facilities might allow captive Filipino CM to expand TRIMCyp-minor (NE) haplotype in their population. Although it might also be hypothesized that the difference in the frequency of these haplotypes between wild-caught and captive animals was a consequence of the founder effect, the fact that more than 100 animals were introduced from wild (wild-caught animals) to breeding and rearing facility (captive animals) by dividing into several times suggests that the difference in the frequency of TRIMCyp haplotype may not be due to founder effect.

Since it is assumed that Filipino CM originated from Indonesian CM stocks (Thierry and Abegg, 2002), the fact that Malaysian and Indonesian CMs also possess TRIMCyp-major (DK) implies that this haplotype arose earlier than the migration of Indonesian CM stocks to the Philippine islands. Probably, TRIMCyp-major (DK) appeared in the ancestor of these CMs for some reason. Since only CM but neither PM nor RM possess TRIMCyp (DK) as one of the TRIMCyp haplotypes, it is reasonable to speculate that some pathogen(s) exerted a strong selection pressure on CM during their evolution. After the appearance of TRIMCyp-major (DK), Malaysian CM continued to maintain TRIMCyp-minor (NE) at a frequency of approximately 10% of total TRIMCyp alleles, while Filipino CM might exclude this haplotype. Alternatively, since Filipino CMs are thought to have originated from a small group of Indonesian CMs (Blancher et al., 2008), the limited prevalence of TRIMCyp-minor (NE) in wild Filipino CMs might be due to a founder effect. Unfortunately, we were unable to place a statistically meaningful value on the prevalence of the TRIMCyp-minor (NE) allele in wild Indonesian CM, since the sample size was too small. In the case of Malaysian CM TRIMCyp, the high frequency of the TRIMCyp-major (DK) allele suggests that it is preferable to posses this haplotype in their habitat. From this point of view, it will be of interest to consider why TRIMCyps of PMs and RMs are NE rather than DK type. In particular, the habitats of PM partially overlap with those of CM, except for the Java and Philippine islands (Thierry and Abegg, 2002). As Dietrich et al. proposed (Dietrich et al., 2010), it is likely that TRIMCyp evolved in the common ancestor of Asian macaques since TRIMCyp is present in both the silenus group, which includes PM, and the fascicularis group, which includes RM and CM. Furthermore, Ylinen et al. speculated that although the CypA sequence that has been retrotransposed into the macaque *TRIM5* locus is expected to be identical to the inherent CypA sequence, an arginine-to-histidine substitution at amino acid 69 may have occurred early in a common ancestor of Asian macaques. This may have been advantageous in that it helped to expand the spectrum of antiviral activity (Ylinen et al., 2010). This group further speculated that TRIMCyp (NE) arose in PMs and RMs independently; however, it is possible that TRIMCyp (NE) arose in the common ancestor of Asian macaques, since TRIMCyp (NE) is also present in CMs (Table 2). It is reasonable to imagine that the ancestors of PMs and RMs might fix TRIMCyp (NE) in order to protect themselves from invasion by TRIMCyp (NE)-sensitive pathogens. Specifically, the fact that PMs exclusively possess TRIMCyp (NE) instead of TRIM5α or TRIMCyp (DK) implies the importance of maintaining this *TRIM5* genotype in their habitat. Otherwise, the founder or bottleneck effect might affect the prevalence of TRIMCyp haplotypes in these macaque species. As an alternative hypothesis, TRIMCyp-minor (NE) in CM might be a vestige of an introgression between CMs and RMs with TRIMCyp (NE). In any case, future studies should analyze the prevalence of TRIMCyp in wild CMs by using samples from many regions to verify the correlation of genetic prevalence between wild and captive CMs.

More importantly, these two haplotypes in CM TRIMCyp are reported to show different antiviral activity (Ylinen et al., 2010; Dietrich et al., 2011; Saito et al., 2012). We and other groups reported that TRIMCyp-major (DK) suppresses the replication of HIV-1, but not that of HIV-2. Conversely, it was shown that TRIMCyp-minor (NE) suppresses the replication of HIV-2, but not that of HIV-1. Thus, these haplotypes of TRIMCyp present in CM are expected to show different antiviral activity in nature. It will be of great interest to investigate the pathogens that acted as a selective pressure to alter the prevalence of TRIMCyp haplotypes.

Taken together, we analyzed the geographic and genetic characteristics of TRIMCyp in wild-caught CM for the first time and found (1) a higher frequency of TRIMCyp in the Philippine population as compared to those in other populations; (2) a similar tendency in the frequency of TRIMCyp between wild-caught and captive CM, and (3) a significant difference in the frequency of TRIMCyp-minor (NE) haplotype between captive and wild Filipino CM. These results provide important insights into the prevalence of CM TRIMCyp and increase our understanding of the evolution of antiretroviral host factors in Asian macaques.

### Conflict of interest statement

The authors declare that the research was conducted in the absence of any commercial or financial relationships that could be construed as a potential conflict of interest.
